# Establishment and Application of a Prognostic Risk Score Model Based on Characteristics of Different Immunophenotypes for Lung Adenocarcinoma

**DOI:** 10.3389/fgene.2022.850101

**Published:** 2022-04-25

**Authors:** Hong Gao, Yanhong Liu, Yue Hu, Meiling Ge, Jie Ding, Qing Ye

**Affiliations:** ^1^ Biobank of Nanjing Drum Tower Hospital, The Affiliated Hospital of Nanjing University Medical School, Nanjing, China; ^2^ Department of Pathology, The First Affiliated Hospital of USTC, Division of Life Sciences and Medicine, University of Science and Technology of China, Hefei, China; ^3^ Intelligent Pathology Institute, Division of Life Sciences and Medicine, University of Science and Technology of China, Hefei, China

**Keywords:** lung adenocarcinoma, tumor-infiltrating immune cells, immunophenotypes, prognostic model, immunotherapy

## Abstract

**Objective:** Lung adenocarcinoma (LUAD) is a highly heterogeneous tumor. Tumor mutations and the immune microenvironment play important roles in LUAD development and progression. This study was aimed at elucidating the characteristics of patients with different tumor immune microenvironment and establishing a prediction model of prognoses and immunotherapy benefits for patients with LUAD.

**Materials and Methods:** We conducted a bioinformatics analysis on data from The Cancer Genome Atlas and Gene Expression Omnibus (training and test sets, respectively). Patients in the training set were clustered into different immunophenotypes based on tumor-infiltrating immune cells (TIICs). The immunophenotypic differentially expressed genes (IDEGs) were used to develop a prognostic risk score (PRS) model. Then, the model was validated in the test set and applied to evaluate 42 surgery patients with early LUAD.

**Results:** Patients in the training set were clustered into high (Immunity_H), medium (Immunity_M), and low (Immunity_L) immunophenotype groups. Immunity_H patients had the best survival and more TIICs than Immunity_L patients. Immunity_M patients had the worst survival, characterized by most CD8^+^ T and Treg cells and highest expression of PD-1 and PD-L1. The PRS model, which consisted of 14 IDEGs, showed good potential for predicting the prognoses of patients in both training and test sets. In the training set, the low-risk patients had more TIICs, higher immunophenoscores (IPSs) and lower mutation rates of driver genes. The high-risk patients had more mutations of DNA mismatch repair deficiency and APOBEC (apolipoprotein B mRNA editing enzyme catalytic polypeptide-like). The model was also a good indicator of the curative effect for immunotherapy-treated patients. Furthermore, the low-risk group out of 42 patients, which was evaluated by the PRS model, had more TIICs, higher IPSs and better progression-free survival. Additionally, IPSs and PRSs of these patients were correlated with EGFR mutations.

**Conclusion:** The PRS model has good potential for predicting the prognoses and immunotherapy benefits of LUAD patients. It may facilitate the diagnosis, risk stratification, and treatment decision-making for LUAD patients.

## Introduction

In the past few decades, the morbidity and mortality of lung cancer have increased year after year. According to the latest WHO data, lung cancer, with morbidity and mortality rates of 11.4 and 18.0% respectively, is the leading cause of cancer-related death (Bray et al., 2018). It also remains the most common cancer and leading cause of cancer-related death in China (Wu et al., 2019). Lung adenocarcinoma (LUAD) is the most common histologic subtype of non-small cell lung cancer (NSCLC), accounting for 40% of lung cancer incidence (Chen et al., 2014). For a long time, LUAD has been considered a non-immunogenic tumor with high heterogeneity. However, increasing evidence indicates the occurrence and development of LUAD depend on tumor mutations and are closely related to the tumor immune microenvironment (TIME).

The TIME is a complex assembly of the tumor, immune, stromal, and extracellular components (Schurch et al., 2020). The organization of these components at the cellular and tissue levels plays a crucial role in tumor progression (Binnewies et al., 2018; Junttila and de Sauvage, 2013). Tumor development and the immune system, with several innate and adaptive immune cell subpopulations, some of which show phenotypic plasticity and possess memory, are closely linked. The interactions and balance between them two directly influence immunotherapy response (Charoentong et al., 2017). Tumor-infiltrating immune cells (TIICs) play an important part in the TIME of LUAD (Bussard et al., 2016); however, the specific mechanisms remain controversial. With the development of detection techniques, researchers have found that the activation of TIICs and immune escape occur before lung cancer invasion, and TIICs are significantly associated with the survival rate (Mascaux et al., 2019). Furthermore, with the application of immune checkpoint inhibitors (ICIs) attracting widespread attention, the indispensable role of TIICs in immunotherapy has also become a research focus. The analysis of immunogenomic data by using bioinformatics tools can provide information on the composition, function, and localization of TIICs; predict tumor mutation burden (TMB) and tumor neoantigen; and indicate immunotherapy response (Schumacher and Schreiber, 2015).

Therefore, we conducted immunotyping of patients based on TIICs and constructed a prognostic risk model based on differentially expressed genes of each phenotype to evaluate the prognosis and immunotherapeutic benefits. We hoped to determine the characteristics of patients with different TIME; screen immune-related differentially expressed genes; establish an effective model to predict the benefits of immunotherapy and the prognoses of patients with LUAD.

## Materials and Methods

### Downloading and Preprocessing of Data on mRNA Sequencing and Somatic Mutations

Data on mRNA sequencing (Fragments Per kilobase of exon model per Million mapped fragments, FPKM) and clinical data of LUAD were downloaded from TCGA as the training set for the next-step analysis. The mRNA sequencing (FPKM) and clinical data of GSE101929, GSE50081, GSE41271, and GSE42127 were downloaded from the Gene Expression Omnibus (GEO) platform. The batch effects between GEO datasets were corrected with the R package SVAR (Irizarry et al., 2003). The processed data were used as the test set for the subsequent analysis. The mRNA sequencing and clinical data of GSE13522 and GSE126044 were also downloaded to evaluate the predictive power of the PRS model for an immunotherapeutic response. The somatic mutation data for the training set were downloaded and analyzed using the R package maftools (Mayakonda et al., 2018). The TMBs and mutation rates of LUAD-related driver genes were calculated. The list of driver genes was derived from Integrative Onco Genomics (https://www.intogen.org/search).

### Patient Recruitment and Sample Inclusion

A total of 42 patients (referred as NJDT patients) with stage I or II LUAD, who underwent surgeries in Nanjing Drum Tower Hospital from January 2017 to January 2018 were randomly selected. Paraffin-embedded samples of tumor and normal tissues were collected. Sections of the paraffin-embedded tissues were stained using hematoxylin–eosin and examined by two pathologists. The samples were graded and classified according to the Eighth Edition of TNM Classification for Lung Cancer proposed by IASLC (Goldstraw et al., 2016). mRNA high-throughput sequencing was performed on tumor and matching normal samples, and the FPKM data was used for follow-up analysis.

### Consensus Clustering of TIICs

The Estimation of STromal and Immune cells in MAlignant Tumor tissues using Expression Data (ESTIMATE) algorithm was used to evaluate the stromal and immune components of samples in the training set and the stromal score, tumor purity, and immune score were calculated (Yoshihara et al., 2013). Based on signal sample Gene Set Enrichment Analysis (ssGSEA) using the R packages of gsva (Hanzelmann et al., 2013) and GSEABase (Reimand et al., 2019), 24 types of TIICs were classified (Bindea et al., 2013): innate immunity (natural killer cells [NKs], NK CD56^dim^ cells, NK CD56^bright^ cells, dendritic cells [DCs], activated DCs [aDCs], immature DCs [iDCs], plasmacytoid dendritic cells [pDCs], neutrophils, eosinophils, mast cells, and macrophages) and adaptive immunity (B, T, T helper 1 [Th1], Th2, T gamma delta [Tgd], CD4^+^ T, CD8^+^ T, T central memory [Tcm], T effector memory [Tem], T follicular helper [Tfh], Th17, regulatory T [Treg], and cytotoxic cells). The training set was clustered hierarchically into high (Immunity_H), medium (Immunity_M), and low (Immunity_L) immunophenotype groups. Then, the CIBERSORT algorithm was used to calculate the relative content of each immune cell subset among 22 types of leukocyte subsets (LM22 signature) with 1,000 permutations (Newman et al., 2015). When the *p* value of the output for each subset was <0.05, the relative contents were considered accurate and suitable for further analysis.

### Identification and Enrichment of IDEGs

For genes with multiple probes, the average of the probes was used as the gene expression. The R package limma (Ritchie et al., 2015) was used to identify DEGs between normal and tumor samples (DEGs_NT) in the training set. DEGs between Immunity_H and Immunity_M (DEGs_HM) and DEGs between Immunity_H and Immunity_L (DEGs_HL) groups were also screened in the same method. DEGs were defined by the false discovery rate (FDR) < 0.05 and Log2|FoldChange| > 1. The intersection of DEGs_NT, DEGs_HM, and DEGs_HL was used to determine IDEGs. Differential pathways were enriched using Gene Set Enrichment Analysis (GSEA). With the |normalized enrichment score (NES)| >1, nominal *p* value < 0.05, and FDR <25%, the enrichment was considered significant.

### Establishment and Validation of the Prognostic Risk Score Model

Univariate Cox regression was used to analyze the correlation between IDEGs and overall survival (OS); genes with *p* < 0.05 were screened. Then, the above genes were analyzed by LASSO regression (Gao et al., 2010) and lambda (λ) values were calculated. Based on the λ value, which corresponded to the minimum mean standard error in the cross-validation, variables were obtained and regression coefficients were calculated. The regression coefficients multiplied by the mRNA levels of 14 genes were used to construct the formula. The median risk score in the training set was used as the grouping cut-off value. Patients with a risk score greater than the cut-off value were classified into the high-risk group; the rest were classified into the low-risk group. Meanwhile, the test set was divided into high- and low-risk groups by using the same cut-off value. The OS curves of the patients in the two sets were plotted, and Log-rank test was used to analyze the differences. The receiver operating characteristic curves (ROCs) of OS in the two sets were plotted, and the areas under curves were calculated to evaluate the predictive performance of the model. Multivariate Cox regression analysis was performed to construct nomograms in both sets.

### Clustering Analysis of *de Novo* Somatic Mutation Signatures in the Training Set

The R package SomaticSignatures (Gehring et al., 2015) was used to identify and cluster *de novo* mutation signatures. The number of these signatures was determined by explained variance and residual sum of squares (RSS). The best number of *de novo* signatures was chosen for clustering. De novo signatures were then compared to 30 curated signatures in the Cancer Gene Census (COSMIC) by using cosine similarity (Cui et al., 2020), Cochran-Armitage trend test was used to examine the mutation signature contribution among groups.

### Immunophenoscores

Immunophenoscores (IPSs) were calculated according to the recently published reports (Charoentong et al., 2017; Hakimi et al., 2016). In brief, consensus determinants including 20 single factors and 6 cell types were divided into four categories: effector cells, suppressive cells, MHC-related molecules, and checkpoints or immunomodulators. The Z scores of the determinants included in the particular category were positively weighted with one and negatively weighted with one. The weighted averaged Z score was then calculated by averaging the Z scores within the respective category leading to four values. The IPSs were calculated on an arbitrary scale of 0–10 based on the sum of the weighted average Z scores of the four categories.

### Workflow

**FIGURE 1:**
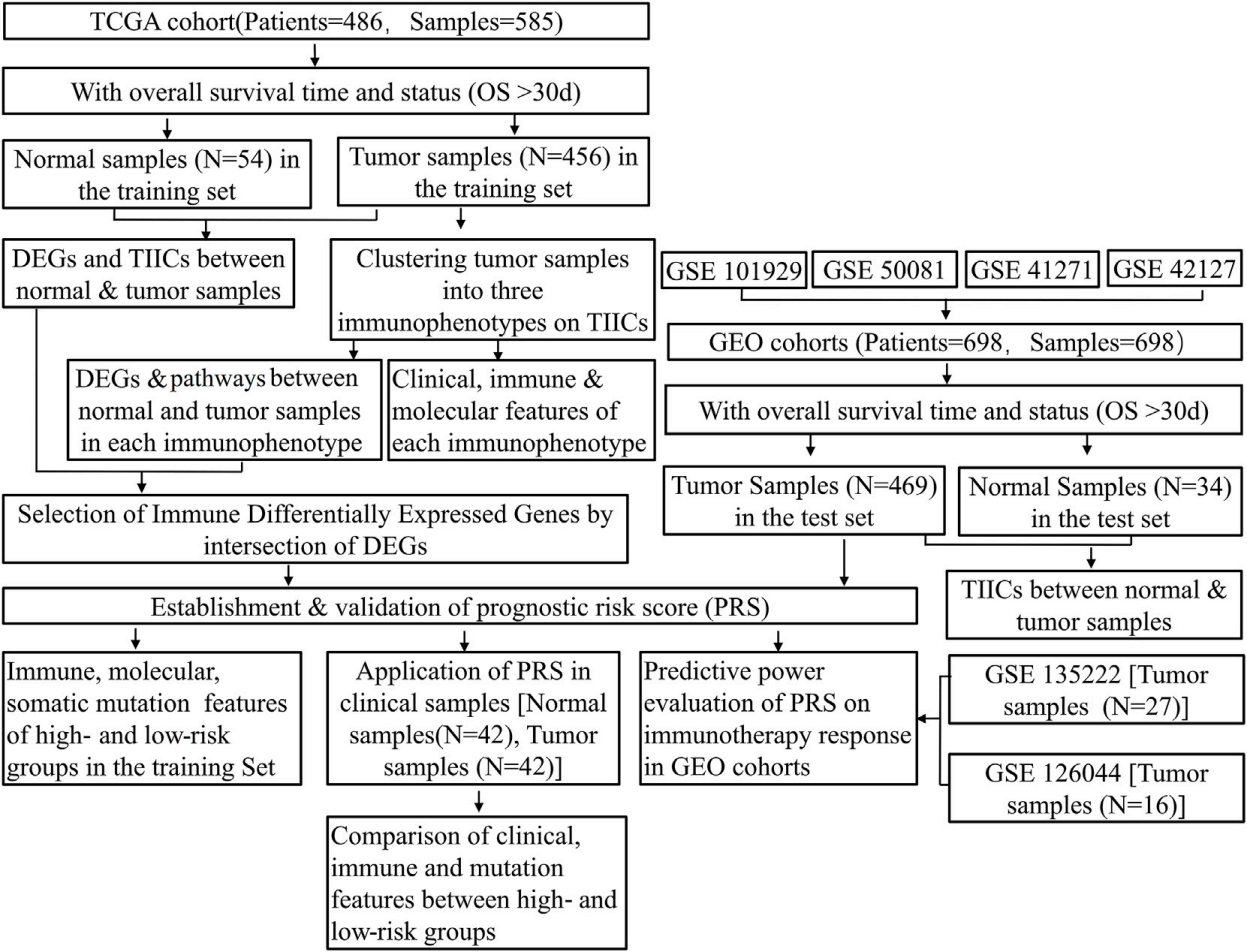
Flowchart of the study protocol. TCGA, The Cancer Genome Atlas; GEO, Gene Expression Omnibus; OS, overall survival; ESTIMATE, Estimation of STromal and Immune cells in MAlignant Tumor tissues using Expression data; TIICs, tumor-infiltrating immune cells; DEGs, differentially expressed genes; PRS, prognostic risk score.

The workflow of this study is shown in Figure 1.

### Statistical Analysis

All statistical analyses were conducted with the R software (version 4.0.2). The Wilcoxon test was used to compare continuous variables in two groups. The Kaplan-Meier plotter was employed to generate survival curves for the subgroups in each dataset. The Log-rank test was used to evaluate significant differences in survival. The Chi-square test or Fisher’s exact test were used to analyze the clinicopathological categorical variables between the different PRS subgroups. Spearman correlation analysis was used to compute the correlation coefficient between indicators. The multiple hypothesis test with the Benjamini–Hochberg method was used to control FDR. All statistical tests were two-sided, and *p* values less than 0.05 were considered statistically significant (∗*p* < 0.05, ∗∗*p* < 0.01, ∗∗∗*p* < 0.001).

## Results

### TIICs Evaluation and Immunotyping

We analyzed the contents of 24 types of TIICs in both sets and evaluated the results by principal component analysis (PCA). There were significant differences between tumor and normal samples. The differences could be used to distinguish normal and tumor tissues (Supplementary Figures S1A–C). The contents of adaptive immune cells increased in tumor tissues, while those of innate immune cells decreased (Wilcoxon test, *p* < 0.05) (Supplementary Figures S1B–D).

**FIGURE 2:**
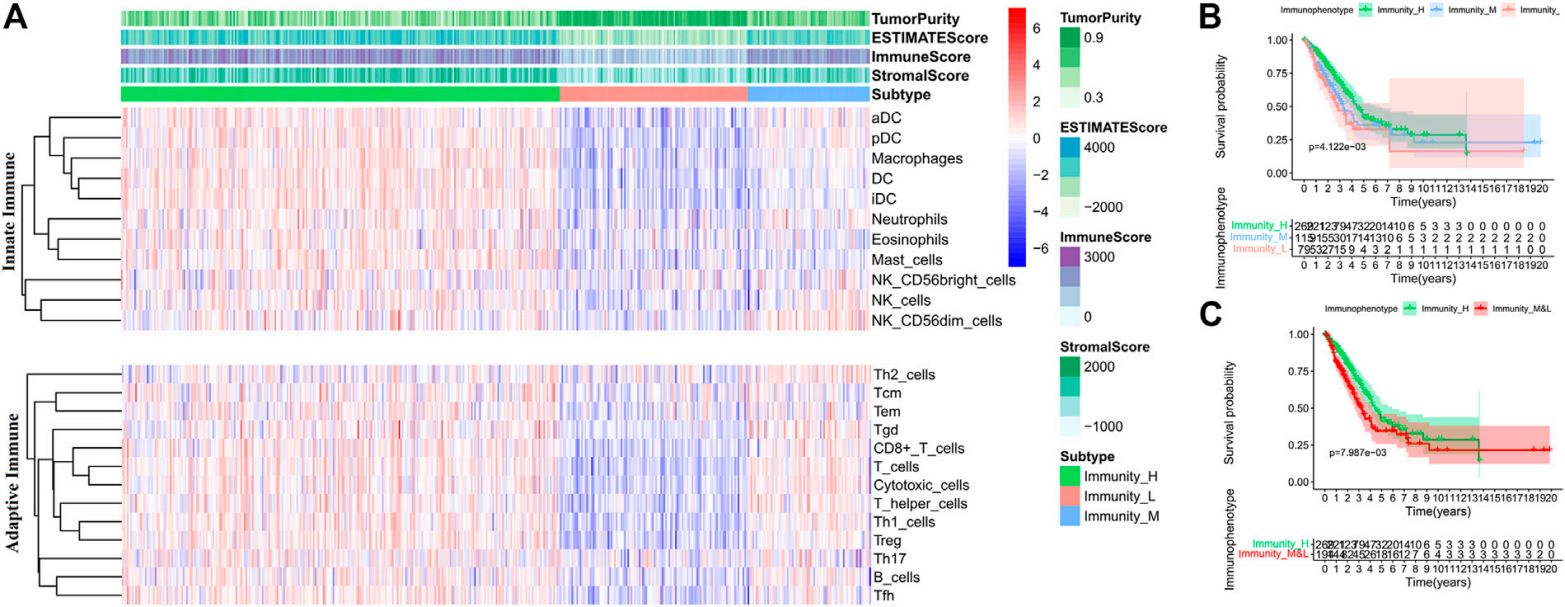
Comparison of TIICs and overall survival (OS) of patients in each immunophenotype in the training set. **(A)** TIIC contents of patients in each immunophenotype. **(B)** Comparisons of OS among the three immunophenotypes. **(C)** Comparisons of OS between Immunity_H and other patients.

Furthermore, the ESTIMATE algorithm was used to evaluate mRNA profiles of tumor samples in the training set. The OS of the patients in the high score (greater than the median value) group based on the immune scores was higher than those of the patients in the low score group, and the intergroup difference was significant (Log-rank test, *p* < 0.05) (Supplementary Figure S2A). It indicates that the prognoses of patients with high immune scores are better than those of the patients with low immune scores. Therefore, hierarchical cluster analysis was performed on the TIICs in tumor samples (Supplementary Figure S2B). According to the immune scores, three clusters were defined as high (Immunity_H), medium (Immunity_M), and low immunophenotypes (Immunity_L) (Figure 2A). The OS of the three immunophenotype groups was statistically different (Log-rank test, *p* < 0.05) (Figure 2B). The patients in the Immunity_H group had the better OS than others (Log-rank test, *p* < 0.05) (Figure 2C). The TIICs in each immunophenotype were further compared. The levels of mature immune cells in the Immunity_L group were the lowest. Almost all innate immune cells in the Immunity_H group were more than those in the Immunity_M group, except Tfh, CD8^+^ T, and Treg cells. These three kinds of cell increased in the Immunity_M group (Wilcoxon test, *p* < 0.05) (Supplementary Figures S3A,B). We also used the CIBERSORT method to quantitate TIICs in each immunophenotype group. Twenty-two types of immune cells were quantified; however, the number of CD4^+^ T naive cells was 0 in all samples. Hence, only 21 types of immune cells were finally analyzed. The contents of most innate immune cells in the Immunity_H group were higher than those in the Immunity_M group (Wilcoxon test, *p* < 0.05) (Supplementary Figure S4A); however, the numbers of CD8^+^ T and Tfh cells in the Immunity_H group were lower than those in the Immunity_M group (Wilcoxon test, *p* < 0.05) (Supplementary Figure S4B).

### Feature Analysis of Different Immunophenotypes in the Training Set

**FIGURE 3:**
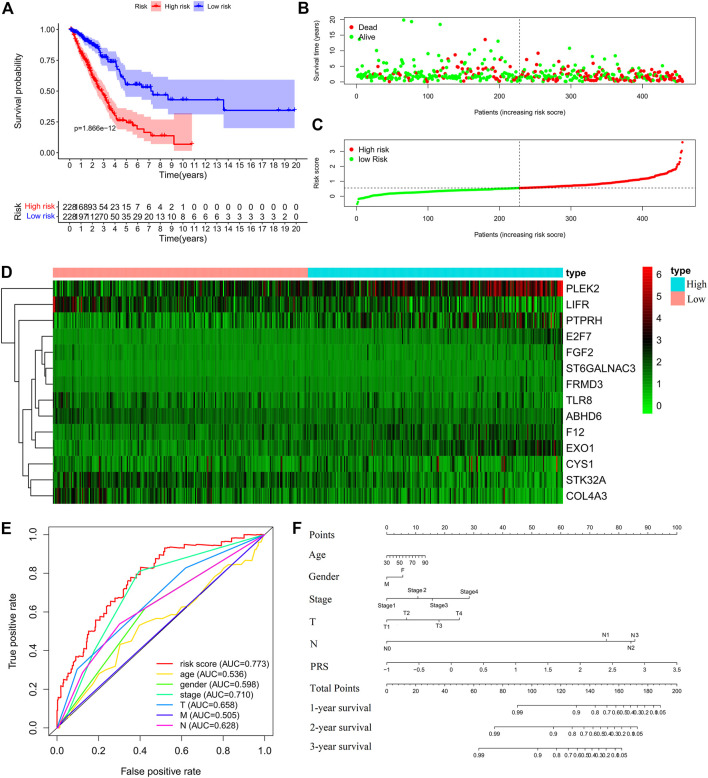
Construction of the prognostic risk score (PRS) model in the training set. **(A)** Comparisons of OS between the high- and low-risk groups. **(B)**. Distribution of survival time of patients with different outcomes; **(C)** Distribution of increasing risk scores in high- and low-risk groups; **(D)** Heatmap of the fourteen-gene expression profiles in high- and low-risk groups; **(E)** ROCs of models with gender, stage, tumor size (T), lymph node metastasis (N), distant metastasis (M), and PRS as variables, respectively; **(F)** Nomogram to predict the 1-year, 2-years and 3-years survival rates of the patients in the training set by using gender, stage, tumor size (T), lymph node metastasis (N), distant metastasis (M), and risk score as variables, respectively.

We analyzed the clinical features of patients in the three immunophenotype groups (Figure 3A, Supplementary Table S1). The proportion of female patients in the Immunity_H group was the highest. The patients in the Immunity_H group had the lowest TMB and the highest IPSs (Wilcoxon test, *p* < 0.05) (Supplementary Figure S5A). We also compared HLA expressions and checkpoints in the three immunophenotype groups. The levels of PD-L1, PD-1, FASL, CTLA4, and CD244 in the Immunity_M group were higher than those in the Immunity_H group (Supplementary Figure S4C). In the Immunity_L group, the expression levels of HLA and checkpoints were lower than those in the Immunity_H group (Wilcoxon test, *p* < 0.05) (Supplementary Figure S4D).

GSEA analysis of the differential enrichment pathways of Kyoto Encyclopedia of Genes and Genomes (KEGG) showed that, the pathways of nucleotide sugar metabolism, DNA stability and autophagy regulation were significantly upregulated (Supplementary Figure S5B), but immune-related pathways and cell adhesion were significantly downregulated of tumor samples in all three immunophenotypes (Supplementary Figure S5C). The Immunity_H group had the most obvious upregulation of metabolism pathways, such as glucose, lipid and water salt metabolism and lysosome pathways. The Immunity_M group was more strongly related to homologous recombination, DNA replication and repair and gene transcription. The Immunity_L group was specifically associated with lowered immune-related pathways, including B and T cell receptor signaling pathways, NK cell mediated cytotoxicity, cytokine receptor interaction and complement-related pathways (Supplementary Figures S5D,E). Thereafter, we intersected DEG_HL, DEG_HM and DEG_NT and obtained 421 IDEGs for subsequent screening (Supplementary Figure S5F). The molecular function and biological processes of these genes covered immune response, regulation of gene silencing, glucolipid metabolism, cell adhesion and blood coagulation (Supplementary Figure S5G).

### Construction and Validation of the PRS Model

Cox regression analysis was performed for the candidate genes among the IDEGs that were specifically associated with OS (Log-rank, *p* < 0.05), followed by LASSO logistic analysis. The most suitable tuning parameters (λ) and coefficients were calculated by cross-validation (Supplementary Figure S6A). Finally, 14 IDEGs were selected to construct the PRS model. The formula was as follows: Prognostic Risk Score = (−0.0461 × *TLR8* mRNA level) + (0.0992 × *FGF2* mRNA level) + (0.0467 × *F12* mRNA level) + (0.3515 × *ST6GALNAC3* mRNA level) + (0.0198 × *PTPRH* mRNA level) + (0.0368 × *EXO1* mRNA level) + (0.0182 × *FRMD3* mRNA level) + (0.1891 × *E2F7* mRNA level) + (−0.1644 × *ABHD6* mRNA level) + (-0.0423 × *STK32A* mRNA level) + (−0.0203 × *COL4A3* mRNA level) + (0.0178 × *PLEK2* mRNA level) + (−0.0222 * *LIFR* mRNA level) + (0.04453 × CYS1 mRNA level). The median score in the training set was considered as the cut-off value, and the patients were divided into high-risk (228 cases) and low-risk (228 cases) groups (Supplementary Table S2). Survival analysis showed that OS (Figures 3A–C), disease-free survival (DFS), progression-free survival (PFS), and disease-specific survival (DSS) (Supplementary Figure S6B) in the high-risk group were significantly worse than those of the low-risk group (Log-rank test, *p* < 0.001). The expression profiles of 14 genes were visualized as a heatmap (Figure 3D). The area under the ROC was 0.773 (Figure 3E). Then, the variables of age, stage, T, M, N, and PRS were analyzed to establish a nomogram for predicting the 3-years survival rate (Figure 3F).

**FIGURE 4:**
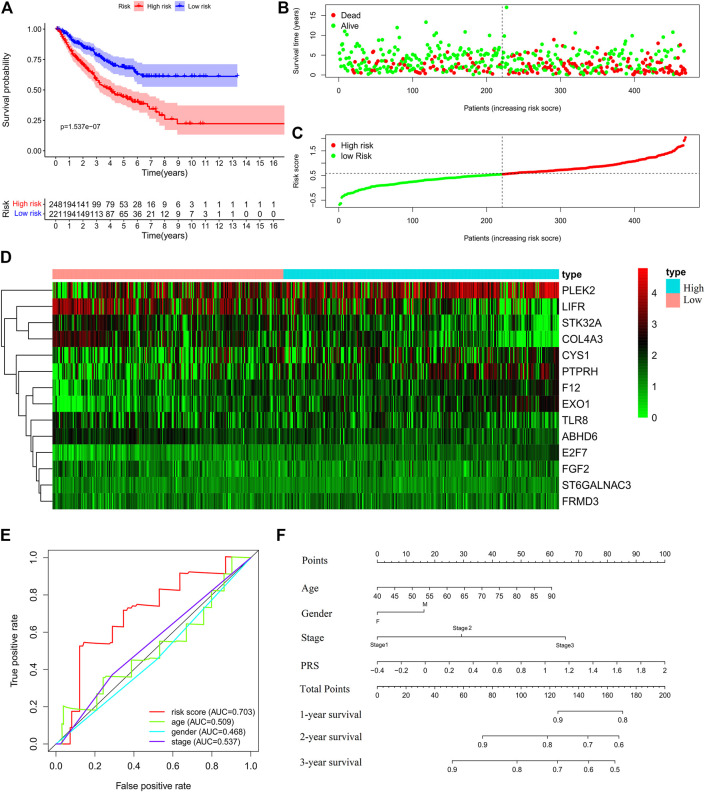
Validation of the prognostic risk score (PRS) model in the test set. **(A)** Comparisons of overall survival (OS) between the high- and low-risk groups. **(B)** Distribution of survival time among patients with different outcomes. **(C)** Distribution of increasing risk scores in high- and low-risk groups. **(D)** Heatmap of the 14-gene expression profiles in high- and low-risk groups. **(E)** ROCs of models with sex, stage, and PRS as variables in the test set s, respectively. **(F)** Nomogram to predict the 1-, 2-, and 3-years survival rates of patients in the test set by using sex, stage, and risk score as variables, respectively.

Further, the PRS model was invalidated in the test set. The above PRS formula, cut-off value, and grouping method were used to divide patients into high-risk (248 cases) and low-risk (221 cases) groups (Supplementary Table S3). The OS of the high-risk group was significantly worse than that of the low-risk group (Log-rank, *p* < 0.001) (Figures 4A–C). The expression profiles of the PRS model genes were visualized as heatmaps (Figure 4D). The area under the ROC was 0.707 (Figure 4E). Then, the variables of age, stage, and risk score were analyzed to establish a nomogram for predicting the 3-years survival rate (Figure 4F).

### Molecular, Immune, and Mutation Features of PRS Subgroups in the Training Set

**FIGURE 5:**
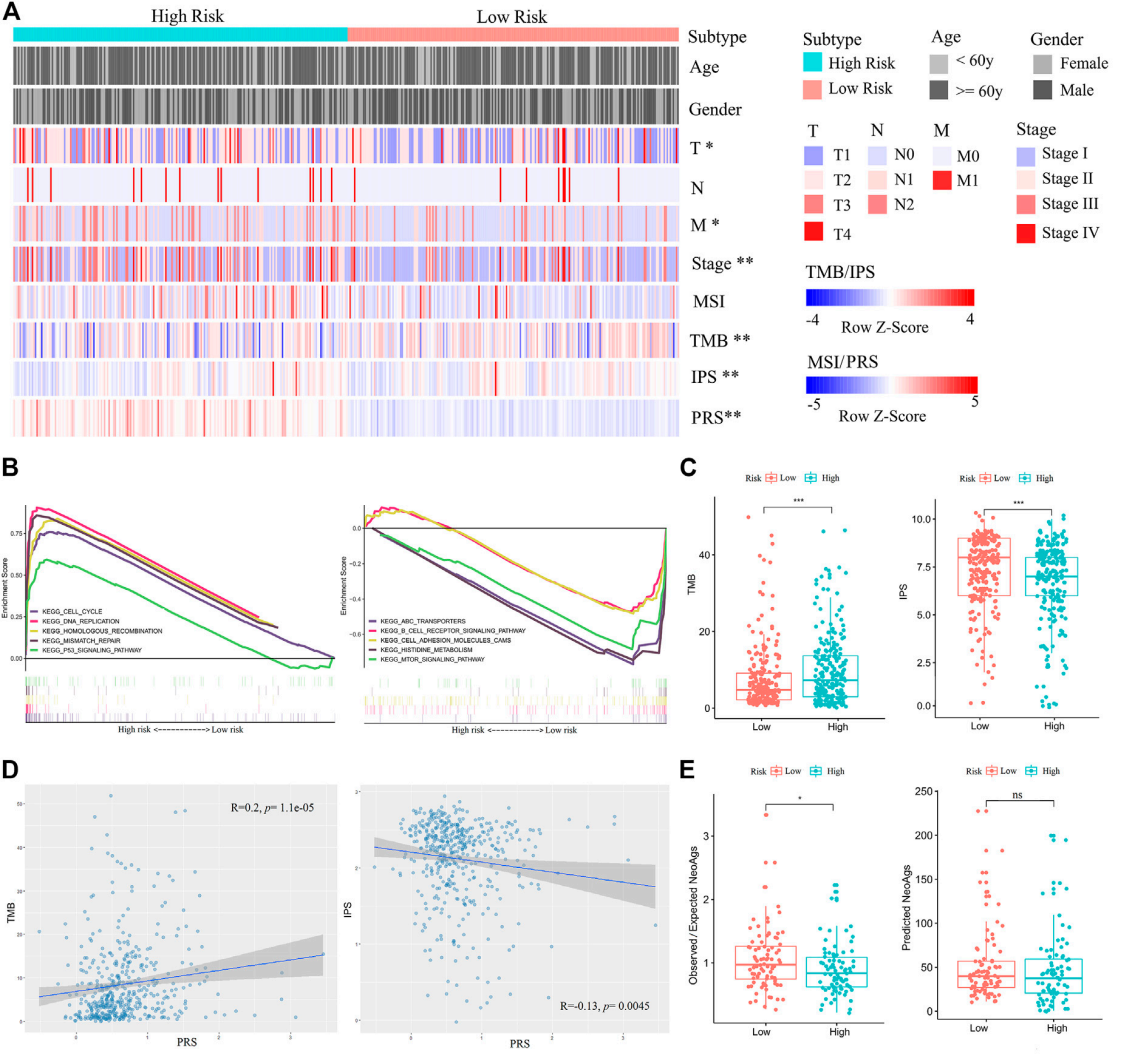
Comparisons of clinical, immune, and molecular features between high- and low-risk patients in the training set. **(A)** Associations of three immunophenotypes with 10 variables. Fisher’s exact test was used for categorical variables: age, sex, pathological stage, tumor size (T), lymph node metastasis (N), and distant metastasis (M). Wilcoxon test was used for continuous variables: MSI, TMB, IPS, and PRS. **(B)** GSEA (C2: curated gene sets, CP: KEGG) showed that the five top pathways upregulated in the high-risk group were cell cycle, DNA replication, homologous recombination, mismatch repair, and p53 signal pathway (left). The five top pathways upregulated in the low-risk group were ABC transporters, B cell receptor signaling pathway, cell adhesion molecules (CAMs), histidine metabolism, and mTOR signaling pathway (right). **(C)** Comparisons of TMBs and IPSs between high- and low-risk patients. **(D)** The positive correlation between TMB and PRS is shown on the left. The negative correlation between IPS and PRS is shown on the right. **(E)** Comparisons of Observed/Expected NeoAgs and Predicted NeoAgs between high- and low-risk patients. The symbol “ns” represents there is no significant difference between the two groups.

After obtaining the reliable PRS model, we analyzed clinical and molecular features of PRS subgroups in the training set. The PRS subgroups showed significant differences in sex, T and N classifications, and stage. The proportion of female, T1, N0, and Stage I patients in the low-risk group was significantly higher than those in the high-risk group (Chi-square test, *p* < 0.05) (Figure 5A, Supplementary Table S4). GSEA analysis on the enrichment pathways of KEGG between the two PRS subgroups showed that pathways of cell cycle, DNA replication, homologous recombination, mismatch repair, p53 signal pathway, which were associated with gene mutation and chromosome instability, were significantly upregulated in the high-risk patients. In contrast, ABC transporters, B cell receptor signaling pathway, cell adhesion molecules (CAMs), histidine metabolism, and mTOR signaling pathway and other immune-related pathways were significantly upregulated in the low-risk patients (Figure 5B). In addition, PRSs of all patients were positively correlated with TMBs (Spearman correlation, *p* < 0.001), and negatively correlated with IPSs (Spearman correlation, *p* < 0.05) (Figures 5C,D) Meanwhile, in combination with literature data, we compared two prediction indicators of neoantigens (Figure 5E): the counts of mutations predicted to yield HLA-binding neopeptides (Predicted NeoAgs) and the ratios of observed versus expected binders per non-silent mutation (Observed/Expected NeoAgs) (Charoentong et al., 2017; Hakimi et al., 2016). The Observed/Expected NeoAgs of the low-risk patients was higher than that of the high-risk ones (Wilcoxon test, *p* < 0.05). Patients in the low-risk group may have more effective neoantigens to promote immunity against tumor and obtain more benefits from immunotherapy.

Then, we also analyzed mutation rates of genes between PRS subgroups in the training set. The integral mutation rate of the high- and low risk group was 92.61 and 78.51%, respectively. Then, mutation frequencies of 42 driver genes associated with LUAD were calculated. Mutation ratios of the driver genes *TP53*, *LRP1B*, *CLIP1*, *EZH2*, *LRIG3*, *PIK3CA*, *RBM10,* and *KRAS* in the high-risk group were higher than those in the low-risk group (Chi-square test, *p* < 0.05) (Supplementary Table S5). These results provide new insights into mutation biomarkers and immunotherapeutic targets. To understand the effects of these mutations on LUAD development, we conducted a cluster analysis and identified 11 signatures of *de novo* mutation (S1-S11) (Supplementary Figures S7A,B). Among them, S2, S3, S4, and S5 were similar to the curated signatures in COSMIC (Supplementary Figure S7C; Supplementary Table S6). S2 was related to DNA mismatch repair deficiency (dMMR), S3 was related to APOBEC (apolipoprotein B mRNA editing enzyme catalytic polypeptide-like), S4 was related to age, and S5 was related to tobacco mutagens. The contributions of S2, S3, S4, S5, S10, and S11 in the high-risk group were higher than those in the low-risk group (Cochran-Armitage trend test, *p* < 0.05) (Supplementary Figure S7D).

### The Predictive Potential of the PRS Model for Immunotherapy Benefits

In the subsequent analysis, we examined the ability of the PRS model to predict the response to immunotherapy in Asian patients. A total of 27 NSCLC patients (GSE135222) who received anti-PD-1/PD-L1 immunotherapy were selected. On the basis of the cut-off values in this study, the patients were divided into high-risk and low-risk groups. Survival analysis showed that low-risk patients had better OS than high-risk ones (Log-rank test, *p* < 0.05, Supplementary Figure S8A). Another dataset of NSCLC patients (GSE126044) who were also treated by anti-PD-1/PD-L1 immunotherapy showed that the PRSs of those who responded to immunotherapy were significantly lower than those who did not respond (Wilcoxon test, *p* < 0.05, Supplementary Figure S8B). The results of these two datasets indicated that the PRS model was also a good predictor for the efficacy of immunotherapy.

### The Prognostic Potential of the PRS Model for NJDT Patients

**FIGURE 6:**
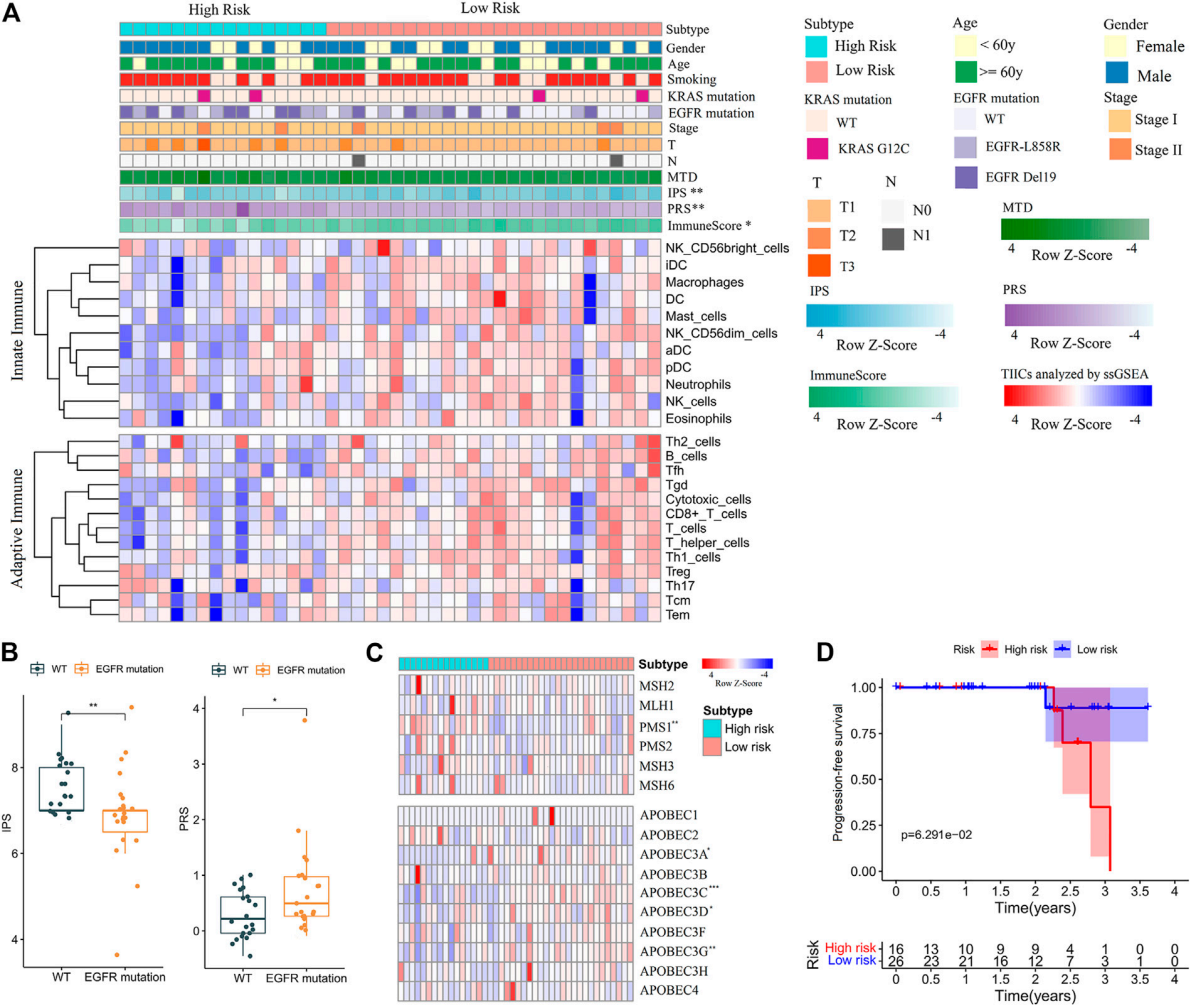
Feature analysis and PFS comparisons of NJDT patients in different PRS subgroups. **(A)** Comparison of the variables between high- and low-risk patients. Fisher’s exact test was used for categorical variables: sex, age, smoking, KRAS mutation, EGFR mutations, tumor size (T), lymph node metastasis (N). Wilcoxon test was used for continuous variables: maximum tumor diameter (MTD), IPS, PRS, Immune Score, and TIICs. **(B)** Comparisons of IPSs and PRSs between EGFR mutation and wild-type patients. **(C)** Heatmaps of MMR and APOBEC genes in high- and low-risk patients. **(D)** Comparisons of PFS between high- and low-risk patients.

Finally, the clinical (Supplementary Table S7) and mRNA sequencing data (Supplementary Table S8) of NJDT patients were analyzed by the PRS model and IPS algorithm (Supplementary Material S1). The PRSs of tumor samples were significantly higher than those of normal samples (Wilcoxon test, *p* < 0.01) (Supplementary Figure S9A); Among the tumor samples, 16 and 26 cases were categorized into the high- and low-risk groups, respectively. When the clinicopathological features were compared, the immune scores (calculated by ESTIMATE) and IPSs of the high-risk group were lower (Figure 6A), and *EGFR* mutations of high-risk patients were more frequent. In addition, patients with *EGFR* mutations had higher PRSs and lower IPSs than wild-type (WT) patients (Wilcoxon test, *p* < 0.05) (Figure 6B). But *KRAS* mutations did not showed the similar phenomenon. These results may suggest that the immunity state against tumor of WT patients was superior to that of mutant patients. Considering the mutation results in the training set, we compared the expression of MMR and APOBEC proteins. *PMS1* was upregulated in the high-risk group, while *APOBEC3A*, *C*, *D* and *G* were upregulated in the low-risk group (Wilcoxon test, *p* < 0.05) (Figure 6C, Supplementary Table S9). Most checkpoints of patients in the low-risk group were higher than those in the high-risk group (Supplementary Figure S9B), and the immune-related pathways in the low-risk group were also upregulated (Supplementary Figure S9C). Furthermore, although the difference between groups was not statistically significant, the low-risk patients had better PFS than high-risk patients (Figure 6D).

## Discussion

TIME has been frequently reported to play an important role in the occurrence and development of LUAD. The evaluation of the dynamic changes in TIME to determine mechanisms underlying tumorigenesis and potential therapeutic targets is of great significance. In our research, the infiltration levels of TIICs in TIME were strongly correlated with patient outcomes. The patients in the Immunity_H group, who had more TIICs and stronger anti-tumor immunoactivity, also had better prognoses. The patients in the Immunity_L group, of whom nearly all mature TIICs were at low levels, did not trigger adequate immune response against LUAD and had worse prognoses. However, it was interesting that the patients in the Immunity_M group, with the worst OS, showed a deficiency of innate immune cells (DC, macrophages and NK cells), but a high level of CD8+T cells. Numerous studies have indicated that NK cells have a definite antitumor effect in the lung cancer (Bhome et al., 2015) and dendritic cells (DCs), as powerful antigen-presenting cells, play important roles in inducing the immune response of CD8+T cells (Hegde et al., 2020; Maier et al., 2020). However, studies on the role of CD8+T cells in TIME have yielded different conclusions. Some reports suggest that the number of these cells is positively correlated with the treatment response and survival of NSCLC patients (Donnem et al., 2015; Rashed et al., 2017). In contrast, recent studies have shown that only approximately 10% of tumor-infiltrating T cells in the TIME of NSCLC patients can recognize surrounding tumor cells, while the rest are “bystander T cells”, which lack response to tumor antigens and are involved in tumor immune escape and progression (Scheper et al., 2019). In addition, Immunity_M showed more Treg cells and higher expressions of CTLA4 and PD-1. The CTLA4 expressed on Treg cells can mediate the downregulation of costimulatory molecules of DCs, reduce DC activation, and enhance the immunosuppressive activity of Treg cells (Chen et al., 2017). The dysfunction of T cells is positively correlated with a high expression of PD-1 (Thommen et al., 2015). These two factors of abnormal T cells and checkpoints might jointly contribute to the worst prognoses of patients in the Immunity_M group, whereas due to the increased CTLA4 and PD-1, they are likely to get more benefits from immunotherapy.

An interesting phenomenon was revealed by analysis of mutation characteristics between high- and low-risk patients in the training set. Somatic mutations in tumor may produce targeted neoantigens recognized by major histocompatibility complex (MHC) (Schumacher and Schreiber, 2015). TMB, as an indicator of somatic mutation in cancer, was lower in the low-risk group, but the predictive amount and proportion of neoantigens were higher. It suggested that although high-risk patients showed more mutations, they did not produce more neoantigens to induce adequate immune response against tumor. The phenomenon may be related to the unsatisfactory infiltration of TIICs and indicate less benefits from immunotherapy. Subsequent analyses on other East Asian patients who received anti-PD-1/PD-L1 immunotherapy repeated the consequence of less benefits from treatment in their high-risk groups. On the other hand, when the PRS model was used to evaluate early-stage LUAD patients, it could not only predict better TIME, but also demonstrate potentials for early LUAD diagnose. Furthermore, *EGFR* mutations were more frequent in high-risk patients, and it suggests that *EGFR* mutation may be associated with immunosuppression in NSCLC (Dong et al., 2017; Gainor et al., 2016). Although we did not detect more details of somatic mutations in these patients, abnormal MMR and APOBEC expressions suggest that there are more mutation differences between high- and low-risk patients, and these differences are also expected to be biomarkers for early diagnosis and prognosis prediction of LUAD.

In previous studies, several immune-related prognostic models of NSCLC based on TCGA datasets have been reported (Chen et al., 2021; Liu et al., 2020; Luo et al., 2020; Song et al., 2020). Some researchers divided TCGA data into the training and test sets, and obtained a prognostic model based on immune genes. The areas under the curves (AUCs) of the model were 0.74 for 3-years OS and 0.70 for 5-years OS in the training set. In the test set, they were 0.676 and 0.523, respectively (Yi et al., 2021a). In our research, AUCs of the PRS model were 0.706 for 3-years OS and 0.710 for 5-years OS in the training set. In the test set, they were 0.636 and 0.631 (Supplementary Figures S6C,D), respectively. The PRS model were performed by the external validation of GEO datasets and had more extensive and stable accuracy and sensitivity in prognostic prediction for LUAD patients. The 14 IDEGs of PRS model are involved in immune cell receptors, inflammatory factors, biological enzymes, gene transcription and blood coagulation. Some of these genes are deeply related to immune environment and immunotherapy. *F12* (coagulation factor XII) regulates a range of innate immune cells (Barbasz and Kozik, 2009; Vorlova et al., 2017), and promotes the differentiation of naive Th cells into TH17 cells (Gobel et al., 2016). *LIFR* (leukemia inhibitory factor receptor subunit alpha) mediates interleukin-6 signaling and is involved in immune regulation (Wang et al., 2020). *TLR8* (Toll-like receptor 8) initiates juvenile T cells, promotes the secretion of various cytokines by DCs and is involved in the regulation of tumor immune microenvironment (Tran et al., 2019), while *FGF2* (fibroblast growth factor 2) is involved in the Wnt/β-catenin, TGF-β and PI3K/Akt pathways to affect the development of LUAD (Dai et al., 2019). TGF-β is an important signaling in promoting cancer metastasis, impairing the functions of immune cells and facilitating immune evasion (Batlle and Massague, 2019). Recently, the anti- TGF-β/PD-L1 bispecific antibody YM101 has reported to effectively overcome treatment resistance and exhibit a superior antitumor activity of non-inflamed tumors (Yi et al., 2021b). The antibody can promote the formation of “hot tumor” in increasing adaptive TIICs and DCs, regulating the ratio of M1/M2, and enhancing cytokine production in T cells. (Yi et al., 2021c). Our research also illustrated the balance of innate and adaptive immune cells and the recognization of T cells by surrounding tumor cells are the keys to improving prognosis and immunotherapy of LUAD. The PRS model may be applied to the predict suitability and efficacy of antibody YM101.

In conclusion, our study provided the risk model, which showed the good predictive ability for the prognosis and therapeutic benefits of LUAD. The exploration based on immunotyping revealed more immune characteristics and molecular mechanisms related to prognosis, and laid a foundation for further research on diagnosis, immunotherapy and drug development. Nevertheless, this study had many limitations, we will further improve the applicability of the PRS model for domestic patients, and conduct more biological experiments to verify the functions and pathways of IDEGs. We hope that our research will facilitate the diagnosis, risk stratification, prognostication, and treatment decision-making for LUAD patients.

## Data Availability

The datasets presented in this study can be found in online repositories. The names of the repository/repositories and accession number(s) can be found below: CNGBdb, CNP0002665.

## Supplementary Material

Supplementary Figure S1 Twenty-four types of TIICs between normal and tumor samples were analyzed in the training and test set. Principal component analysis (PCA) of 24 TIICs between normal and tumor samples in the training set **(A)** and test set **(B)**. The level comparison of 24 TIICs between normal and tumor samples in the training set **(C)** and test set **(D)**

Supplementary Figure S2 OS comparison and hierarchical cluster analysis of TIICs of different groups in the training set. **(A)** OS comparisons of patients in the low- and high-level groups for immune score (left), stromal score (middle), and tumor purity (right). **(B)** Hierarchical cluster analysis of patients in the training set based on the TIICs. Immunity_H group in green, Immnuty_M group in red and Immnuty_L group in blue were shown.

Supplementary Figure S3 Comparison of the levels of 24 TIICs of tumor samples in each immunophenotype. **(A)** The levels of the innate immune cells were compared among three different immunophenotypes. **(B)** The levels of the adaptive immune cells were compared among three different immunophenotypes. The symbol “ns” represents there is no significant difference between the two groups.

Supplementary Figure S4 Comparison of the levels of 21 immune cells and expressions of checkpoints and HLA-related genes in each immunophenotype. **(A)** The levels of 21 immune cells analyzed by CIBERSORT were compared between Immunity_H and Immunity_M. **(B)** The levels of 21 immune cells analyzed by CIBERSORT were compared between Immunity_H and Immunity_L. **(C)** The expressions of checkpoints and HLA-related genes were compared between Immunity_H and Immunity_M. (B) The expressions of checkpoints and HLA-related genes were compared between Immunity_H and Immunity_L. The symbol “ns” represents there is no significant difference between the two groups.

Supplementary Figure S5 Comparisons of clinical and molecular features and immunophenotypic differentially expressed genes (IDEGs) among the three immunophenotypes in the training set. **(A)** Association of the three immunophenotypes with 10 variables. Fisher’s exact test was used for categorical variables: age, gender, pathological stage, tumor size (T), lymph node metastasis (N) and distant metastasis (M); Kruskal-Wallis test was used for continuous variables: MSI, TMB, IPS and Risk Score. **(B)** GSEA (C2: curated gene sets, CP: KEGG) revealed the pathways that were significantly upregulated in various immunophenotypes, compared with normal samples. **(C)** GSEA (C2: curated gene sets, CP: KEGG) revealed the pathways that were significantly downregulated in various immunophenotypes, compared with normal samples. **(D)** Comparison of significant enrichment pathways in tumor samples between Immunity_H and Immunity_M **(E)** Comparison of significant enrichment pathways in tumor samples between Immunity_H and Immunity_L **(F)** 421 immunophenotypic differentially expressed genes (IDEGs) were obtained by intersection of DEGs_NT, DEGs_HM and DEGs_HL. **(G)** Gene Ontology (GO) analysis of IDEGs

Supplementary Figure S6 Construction of prognostic risk score (PRS) model analyzed by LASSO regression and comparison of DFS, DSS, and PFS between the high- and low-risk patients in the training set. **(A)** Tuning parameter (λ) and deviance in the LASSO regression (left). The partial likelihood deviance was plotted versus log (λ). The dotted vertical lines were drawn at the optimal values by using the minimum and 1-SE criteria. Fourteen features with the smallest binomial deviance were selected. LASSO coefficient profiles of texture features (right). Each line represented a variable with the regression coefficient on the vertical axis and the logarithm of λ on the abscissa. A 10-fold cross-validation was used in the log (λ) sequence to select 14 variables with non-zero coefficients. **(B)** Comparisons of disease-free survival (DFS) (left), disease-specific survival (DSS) (middle), and progression-free survival (PFS) (right) of high- and low-risk patients in the training set. **(C)** Time-dependent receiver operating characteristic curves of 3- and 5-year survival in the training set (left) and test set (right).

Supplementary Figure S7 Identification and comparison of de novo mutational signatures in the training set. **(A)** Upper image, residual sum of square (RSS) of the signature number selection. Lower image, percentage of variance explained in the signature number selection. **(B)** Cosine similarity between 30 cosmic signatures (horizontal axis) and 11 de novo signatures (vertical axis) in the training set. **(C)** Contributions of point mutations of de novo mutation signatures (S1−S11) in the training set. **(D)** Comparison of the contributions of de novo mutation signatures (S1−S11) between the high- and low-risk groups

Supplementary Figure S8 Prediction of the prognostic risk score (PRS) model for immunotherapy benefits of patients. **(A)** Comparisons of overall survival (OS) between the high- and low-risk patients in GSE135222. **(B)** Comparisons of PRSs of the responders and non-responders treated by immunotherapy in GSE126044.

Supplementary Figure S9 Analysis of PRSs, checkpoints, HLA-related genes, and enrichment pathways of NJDT patients. **(A)** Comparison of PRSs between normal and tumor samples of NJDT patients. **(B)** The expressions of checkpoints and HLA-related genes were compared between high- and low-risk patients. **(C)** GSEA (C2: curated gene sets, CP: KEGG) showed that the five top pathways (antigen processing and presentation, cell adhesion molecules [CAMs], chemokine signaling pathway, cytokine receptor interaction, and natural killer cell-mediated cytotoxicity) were unregulated in the low-risk group. The symbol “ns” represents there is no significant difference between the two groups.
