# Supplementary figures and images for: Establishment and Application of a Prognostic Risk Score Model Based on Characteristics of Different Immunophenotypes for Lung Adenocarcinoma
Source: Front Genet. 2022 Apr 25;13:850101. doi: 10.3389/fgene.2022.850101 (PMC9081571; doi:10.3389/fgene.2022.850101)

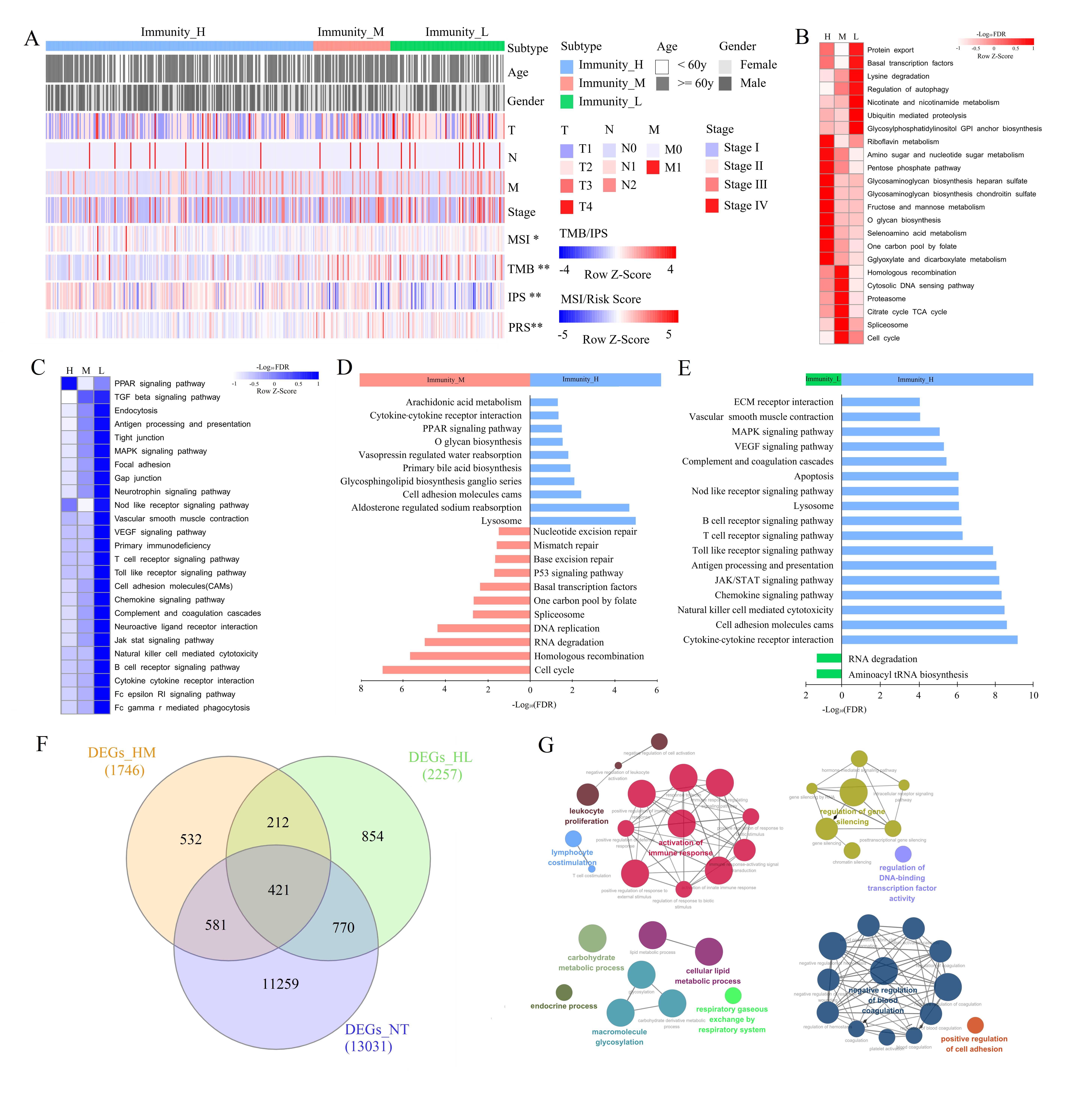

Supplement: Supplementary file 1 [file Image6.TIF]

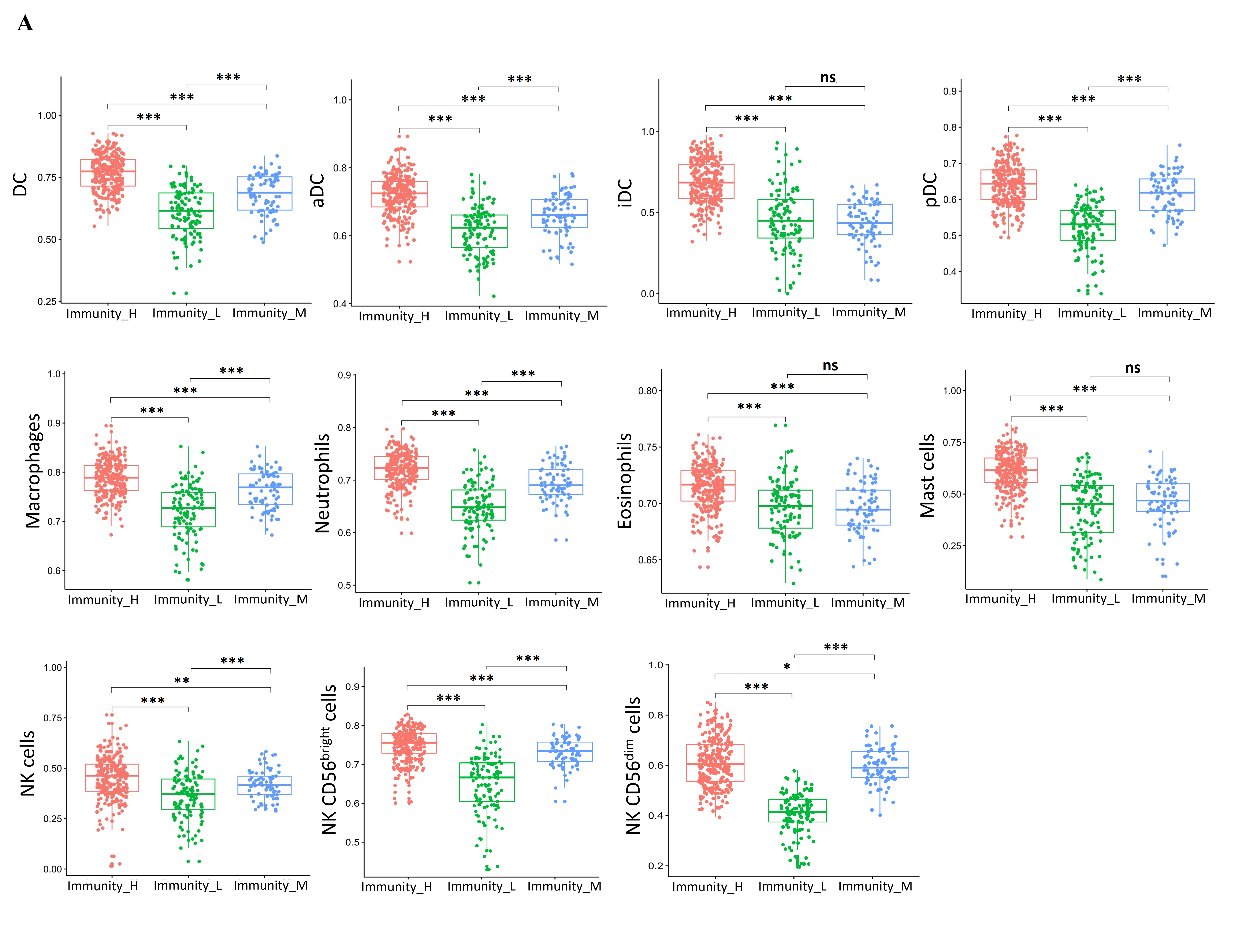

Supplement: Supplementary file 2 [file Image3.TIF]

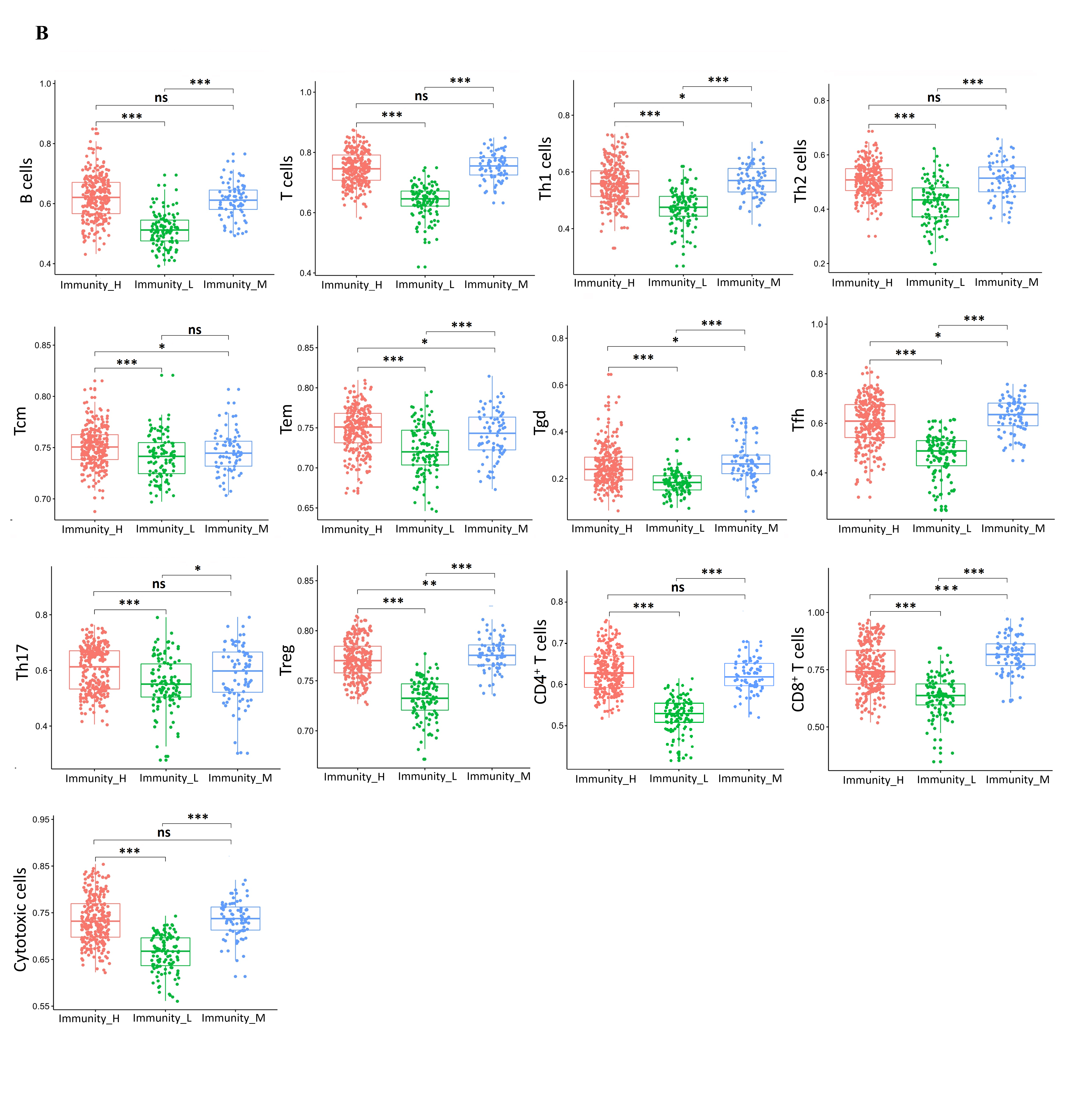

Supplement: Supplementary file 3 [file Image4.TIF]

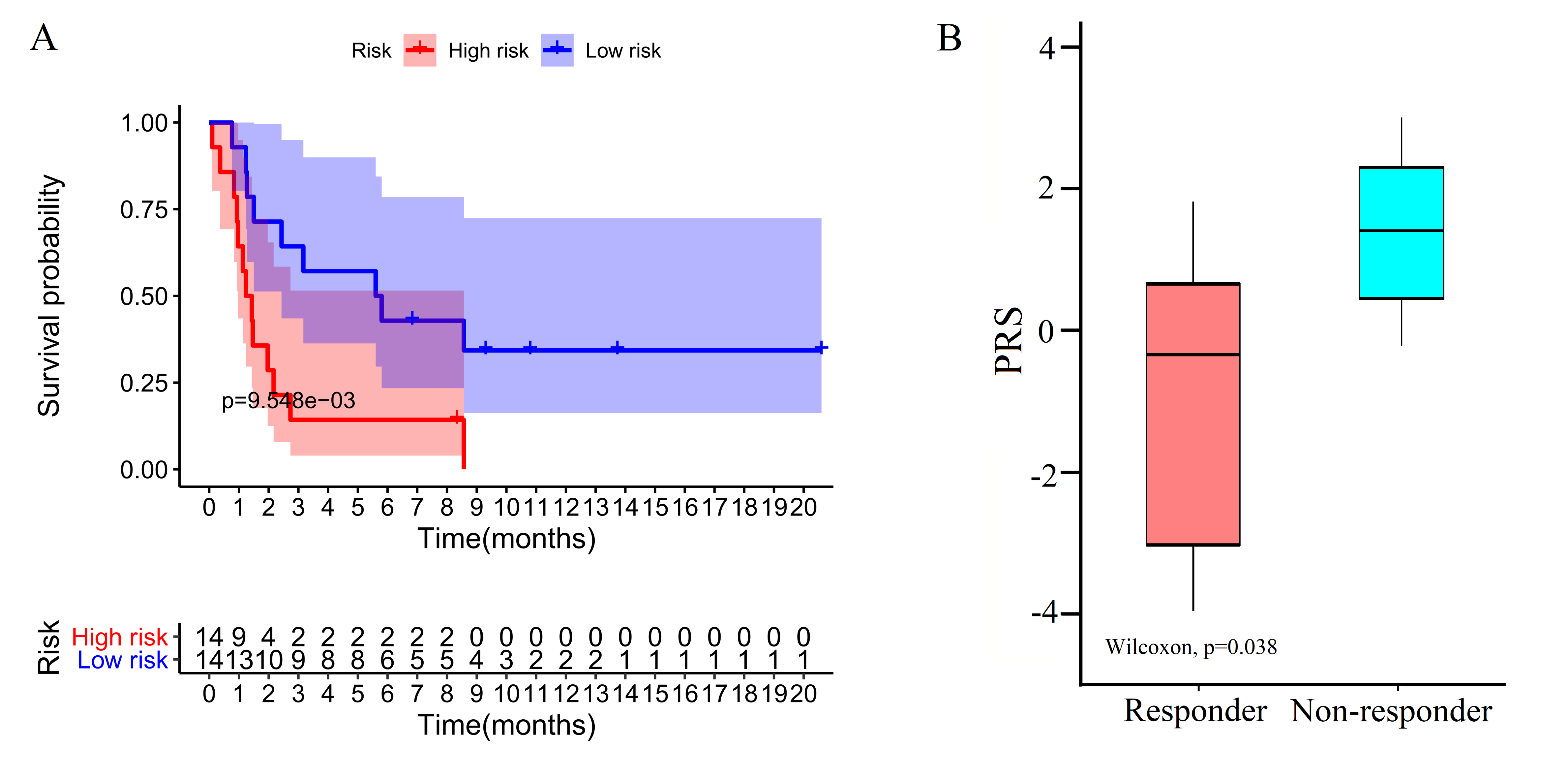

Supplement: Supplementary file 4 [file Image9.TIF]

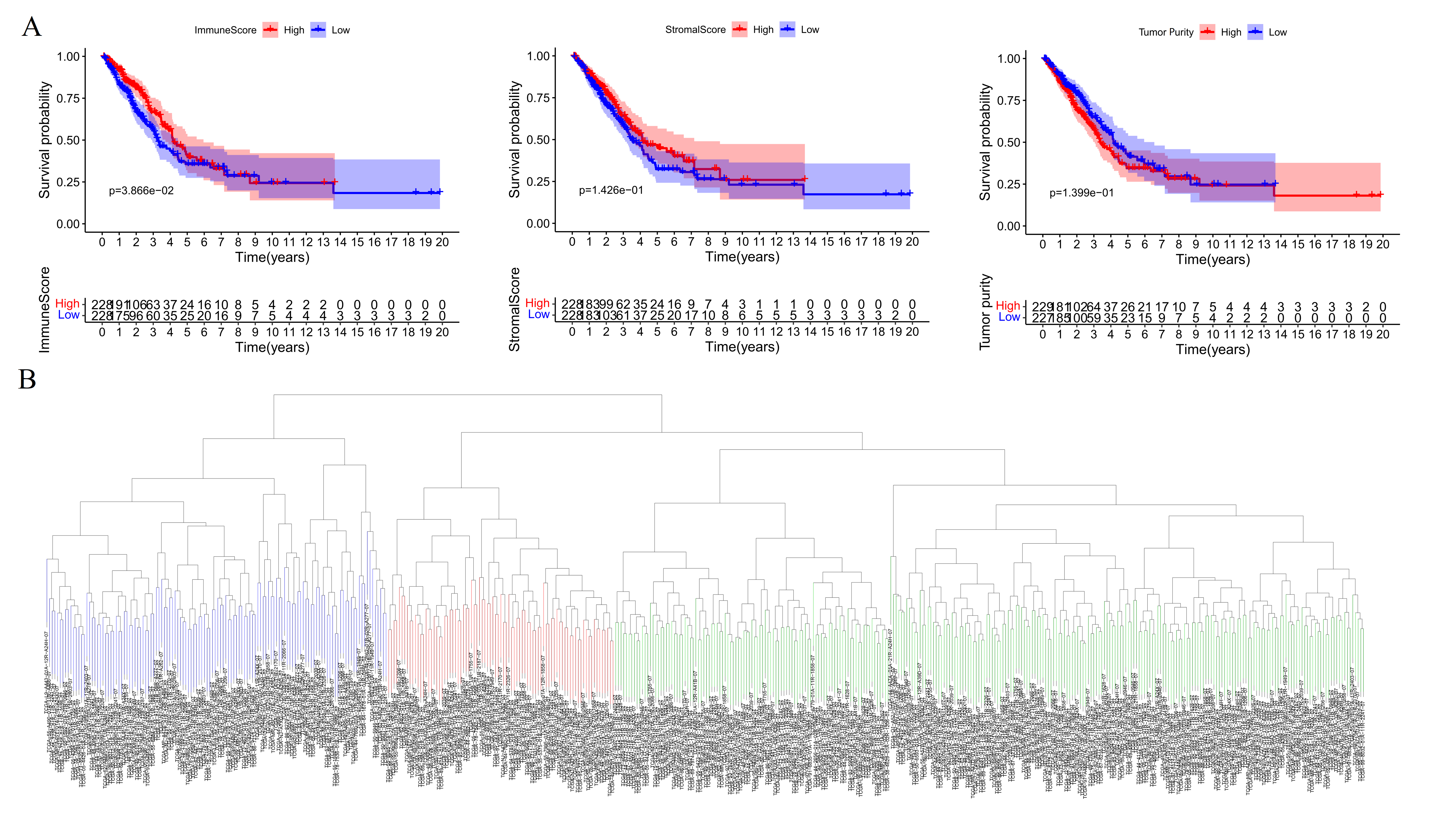

Supplement: Supplementary file 5 [file Image2.TIF]

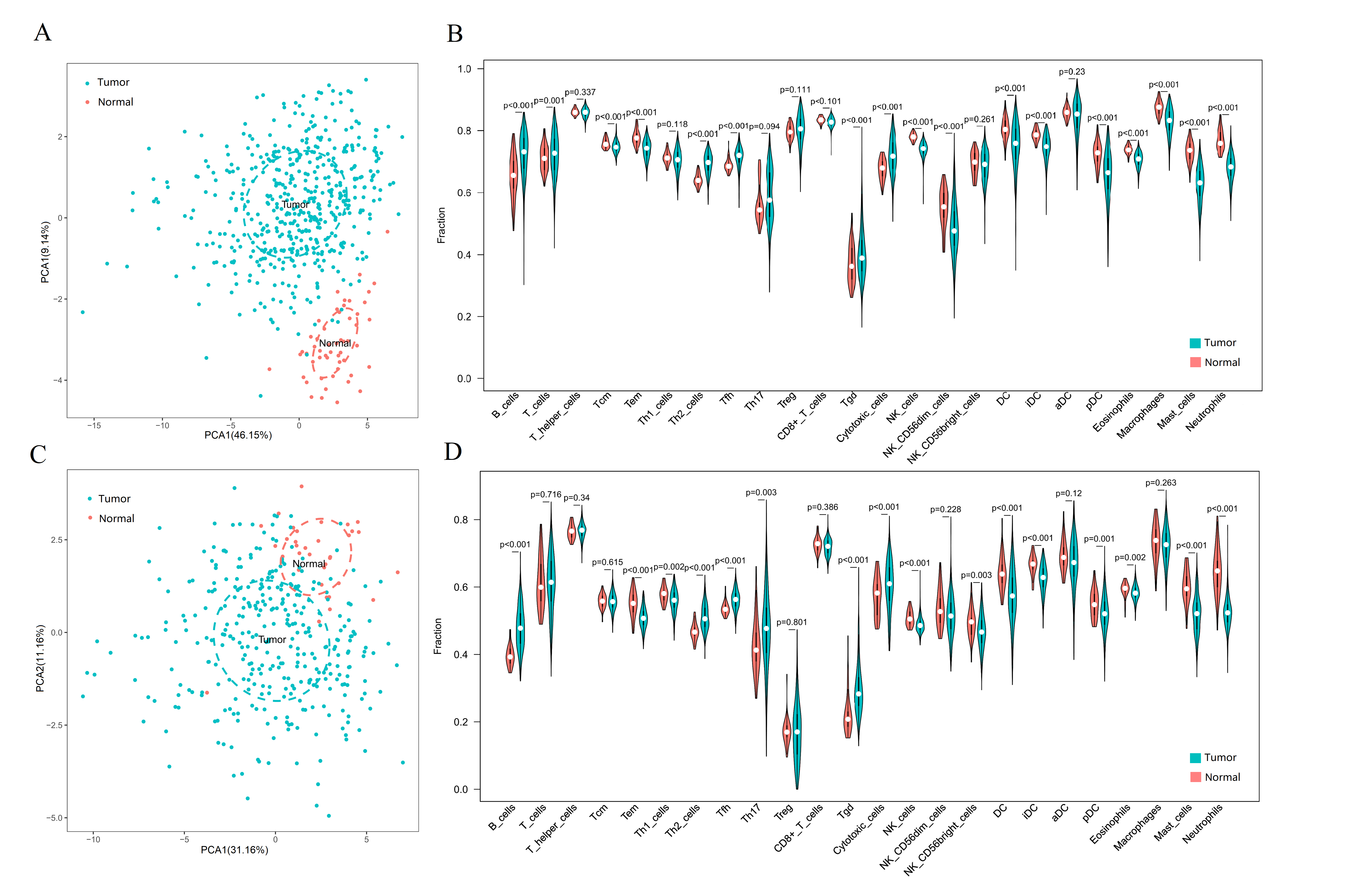

Supplement: Supplementary file 6 [file Image1.TIF]

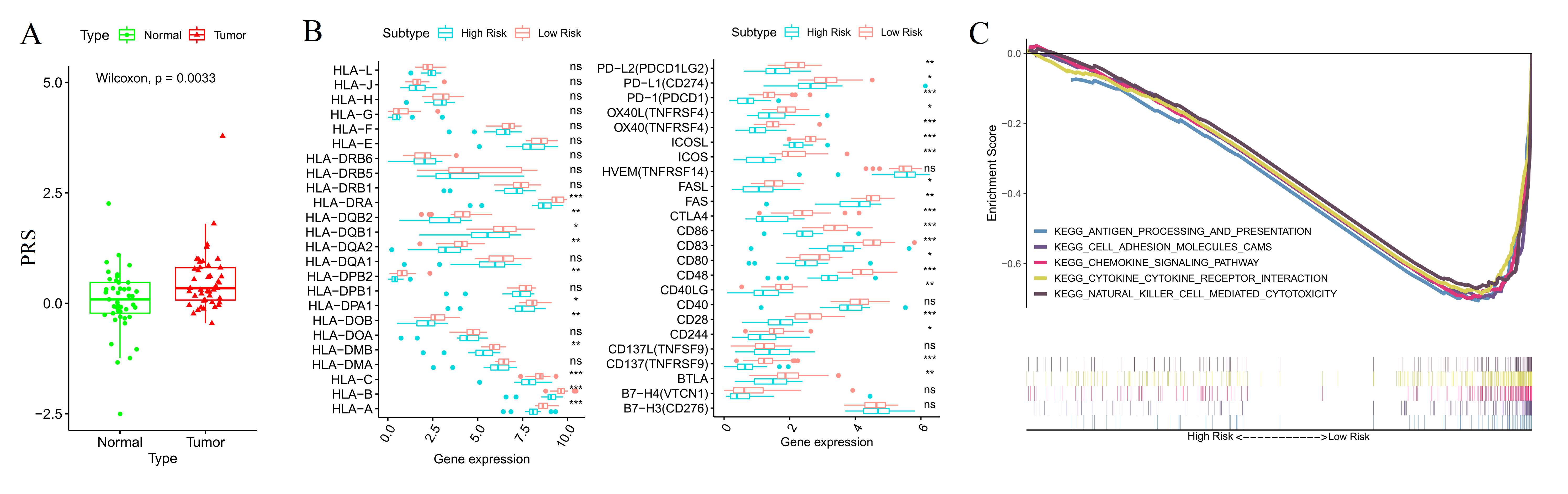

Supplement: Supplementary file 7 [file Image10.TIF]

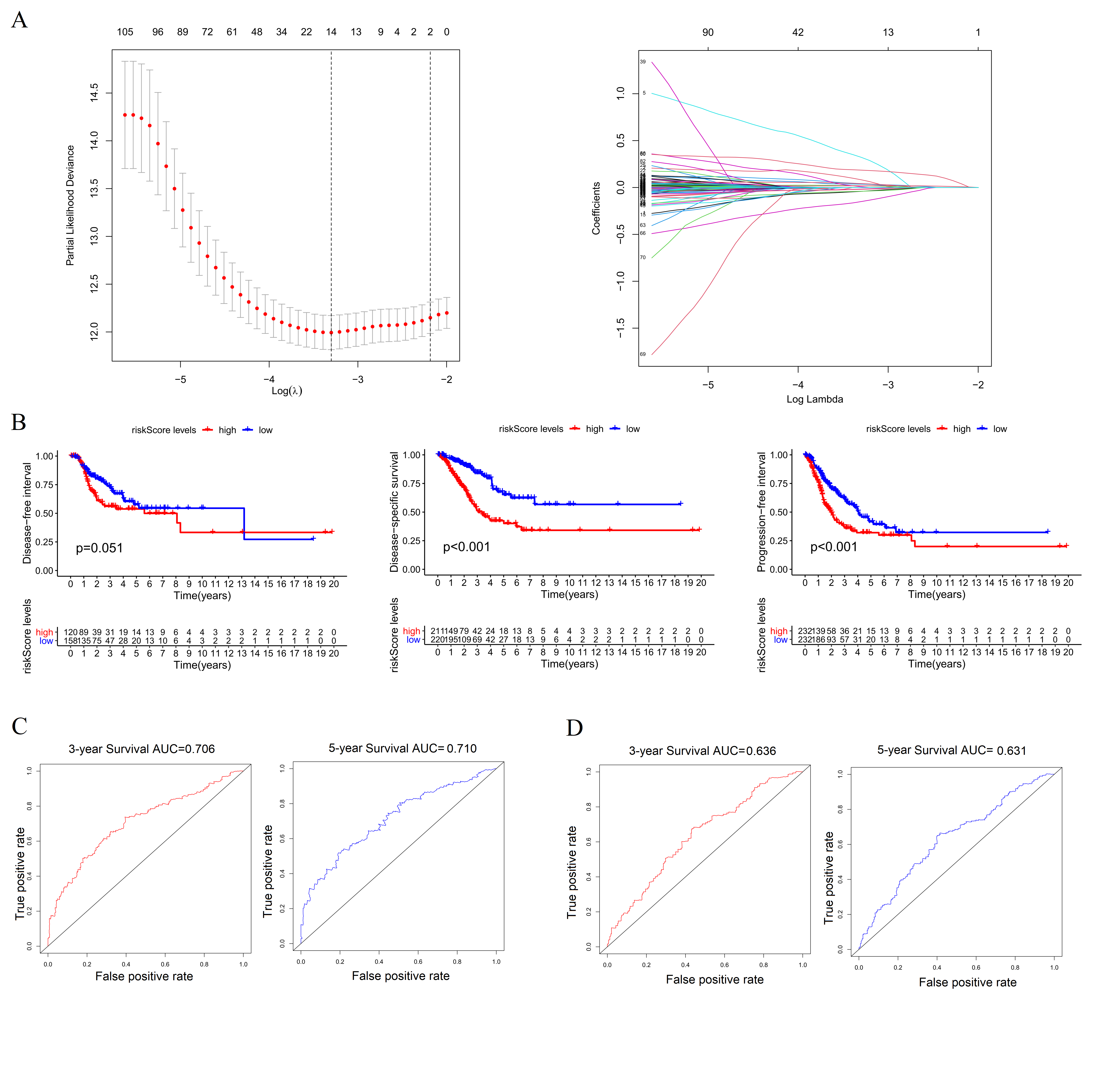

Supplement: Supplementary file 8 [file Image7.TIF]

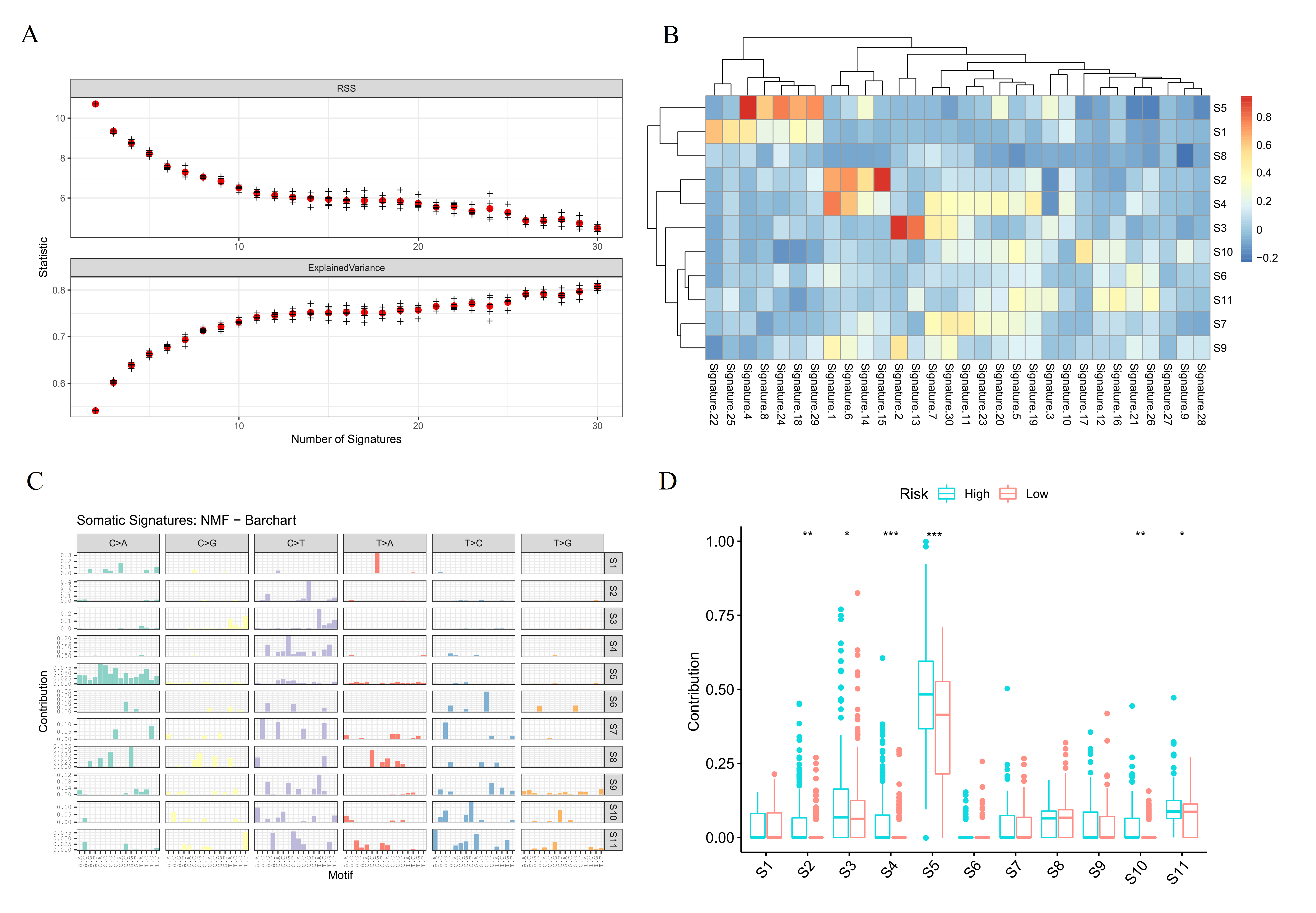

Supplement: Supplementary file 10 [file Image8.TIF]

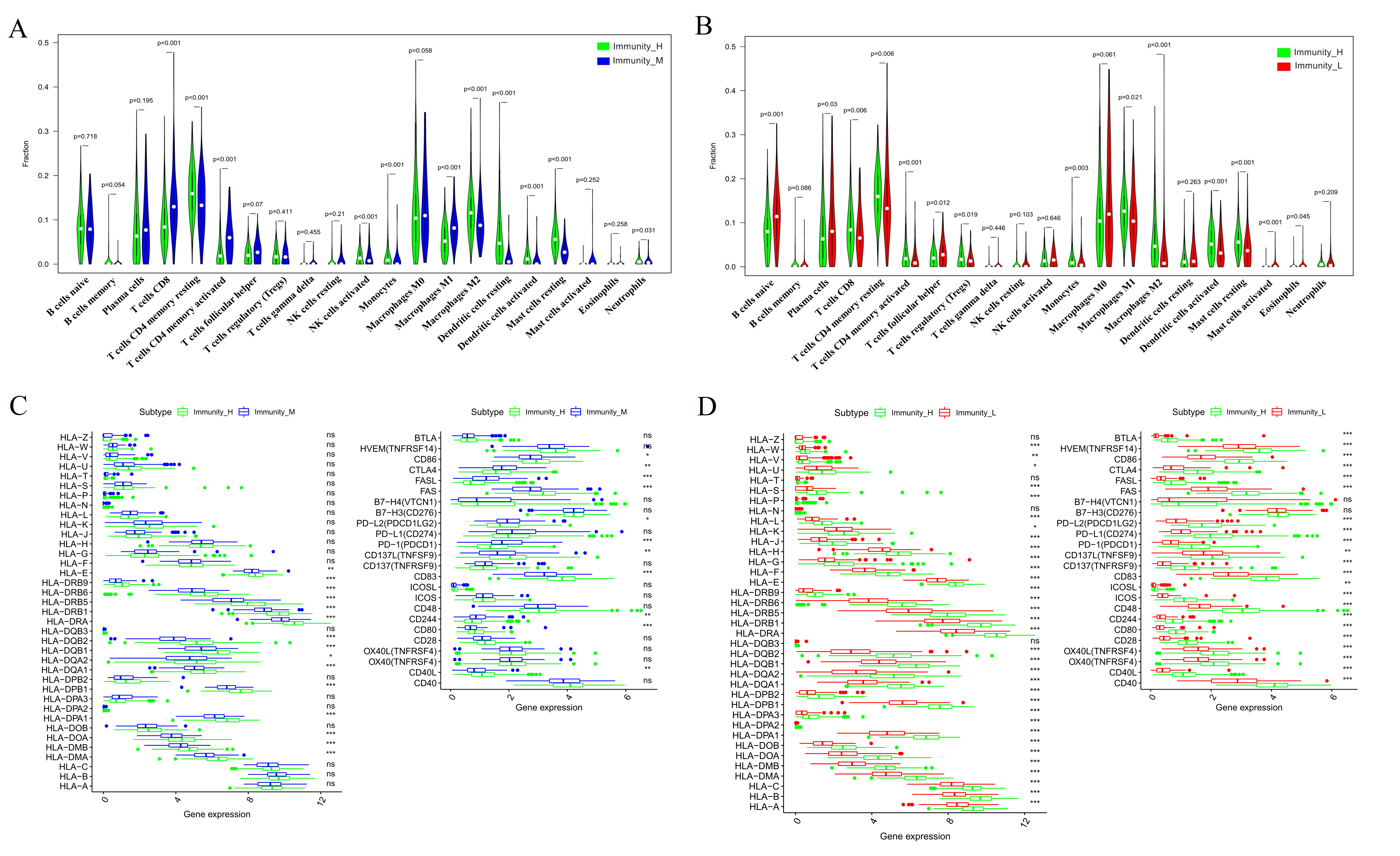

Supplement: Supplementary file 11 [file Image5.TIF]
